# Plasmonic coupling in closed-packed ordered gallium nanoparticles

**DOI:** 10.1038/s41598-020-61090-3

**Published:** 2020-03-06

**Authors:** S. Catalán-Gómez, C. Bran, M. Vázquez, L. Vázquez, J. L. Pau, A. Redondo-Cubero

**Affiliations:** 1 Grupo de Electrónica y Semiconductores, Departamento de Física Aplicada, Universidad Autónoma de Madrid, Cantoblanco, E-28049 Madrid, Spain; 2 Instituto de Ciencia de Materiales de Madrid, Consejo Superior de Investigaciones Científicas (ICMM-CSIC), Cantoblanco, E-28049 Madrid, Spain

**Keywords:** Nanophotonics and plasmonics, Nanoparticles, Synthesis and processing, Surface patterning

## Abstract

Plasmonic gallium (Ga) nanoparticles (NPs) are well known to exhibit good performance in numerous applications such as surface enhanced fluorescence and Raman spectroscopy or biosensing. However, to reach the optimal optical performance, the strength of the localized surface plasmon resonances (LSPRs) must be enhanced particularly by suitable narrowing the NP size distribution among other factors. With this purpose, our last work demonstrated the production of hexagonal ordered arrays of Ga NPs by using templates of aluminium (Al) shallow pit arrays, whose LSPRs were observed in the VIS region. The quantitative analysis of the optical properties by spectroscopic ellipsometry confirmed an outstanding improvement of the LSPR intensity and full width at half maximum (FWHM) due to the imposed ordering. Here, by engineering the template dimensions, and therefore by tuning Ga NPs size, we expand the LSPRs of the Ga NPs to cover a wider range of the electromagnetic spectrum from the UV to the IR regions. More interestingly, the factors that cause this optical performance improvement are studied with the universal plasmon ruler equation, supported with discrete dipole approximation simulations. The results allow us to conclude that the plasmonic coupling between NPs originated in the ordered systems is the main cause for the optimized optical response.

## Introduction

The interaction between metallic nanoparticles (NPs) and the electromagnetic radiation has constituted the driving force for the studies of the light-matter interaction during the last decades^1–3^. Due to their free electrons, the metallic NPs are capable to concentrate and amplify the electric near-field in the vicinities of their surfaces. The electron oscillations (plasmons) resonate with light at a certain frequency that is commonly known as the localized surface plasmon resonance (LSPR). This frequency strongly depends on the NP size, shape, contact angle, environment and, especially, on the metal type^4^. For instance, in the most studied elements, silver (Ag) and gold (Au), their LSPRs are quite restricted to the visible (VIS) region^5^ due to their low losses and interband transitions in the ultraviolet (UV) region.

Thus, during the last years, there has been an effort in the scientific community to search for alternative metals^6–8^. Among others, liquid gallium (Ga) has emerged as an ideal plasmonic candidate^9,10^ since its LSPRs can be tuned from the UV to the infrared (IR) due to the lack of strong interband transitions in this wide region^11^ in contrast to other candidates such as Al^12^, Cu^13^ or Ni^14^. This spectral tunability can be achieved by means of different methods: changing the NP size^15^, contact angle or substrate^16,17^, varying the gallium oxide shell thickness^18^, by hybridization with other plasmonic NPs^19^ or by alloying^9,20^.

In addition to this, the Ga NPs can be grown in a facile, fast and up-scalable method such as Joule-effect thermal evaporation^21,22^. This synthesis technique produces self-assembled hemispherical NPs formed by a liquid Ga core and a self-limiting gallium oxide (Ga_2_O_3_) shell formed when exposed to air that preserves them from the environment without significantly affecting the LSPRs^23^. Interestingly, the liquid nature of the core can be changed by using pulsed light^24,25^.

The mechanism controlling the growth of Ga NPs is coalescence and takes place in a wide range of substrates^26^. Consequently, the typical size distribution obtained is non-uniform being the biggest NPs surrounded by smaller ones^18^. The main disadvantage of these broad size distributions is that the optical performance is reduced since NPs of different sizes resonate at different frequencies resulting in relatively broad and moderate intense LSPRs. Furthermore, the interparticle spacing is not well controlled and plasmonic coupling can hardly take place. Despite this inconvenient, different applications based on Ga NPs have been demonstrated such as surface enhanced Raman spectroscopy (SERS)^27–29^, surface enhanced fluorescence^30,31^, Li-ion batteries^32^, waveguiding^33,34^, optical switching^35,36^, phase-change memories^37^ or the development of biosensors for the detection of different diseases^38,39^.

With the aim to advance in these applications, several recent works have reported different approaches that have improved the size distributions of Ga NPs using IR light^40,41^, corrugated Cu films^29^, polymer nanostructured templates^42^ and even 2D materials^31^. Although in all the cases the NPs were aligned and the size distributions were more homogeneous, NPs of different sizes still coexist. In the literature, one of the most successful approaches for the ordering of NPs has been the use of Al nanostructured templates composed by shallow pit arrays^43^. These substrates are produced by anodized aluminium oxide, commonly known as alumina, that has been widely used for many applications^44^ and nanostructures manufacturing^45,46^. Indeed, ordered distributions of CdTe^47^, In^48^, Ag^49^, Au^50,51^ and Al^52^ NPs have been reported from these templates. Based on this idea, we have previously communicated the production of uniform size distributions of highly ordered Ga NPs^53^. However, that work was restricted to experimental data of a unique NP size whose LSPR was placed in the VIS range. Furthermore, the better optical response caused by the ordering was not deeply investigated.

In this work, we report the fabrication of ordered arrays of Ga NPs of different size by changing the template pattern in order to spread out the LSPRs to a wider range of the electromagnetic spectrum from the UV to the IR. Moreover, we investigate the origin of the optical performance improvement of the experimental results using discrete dipole approximation (DDA) simulations with the aim to represent the different observed scenarios. Lastly, we apply the plasmon ruler equation to the experimental and calculated data in order to evaluate whether plasmonic coupling takes place.

## Results and Discussions

**Figure 1:**
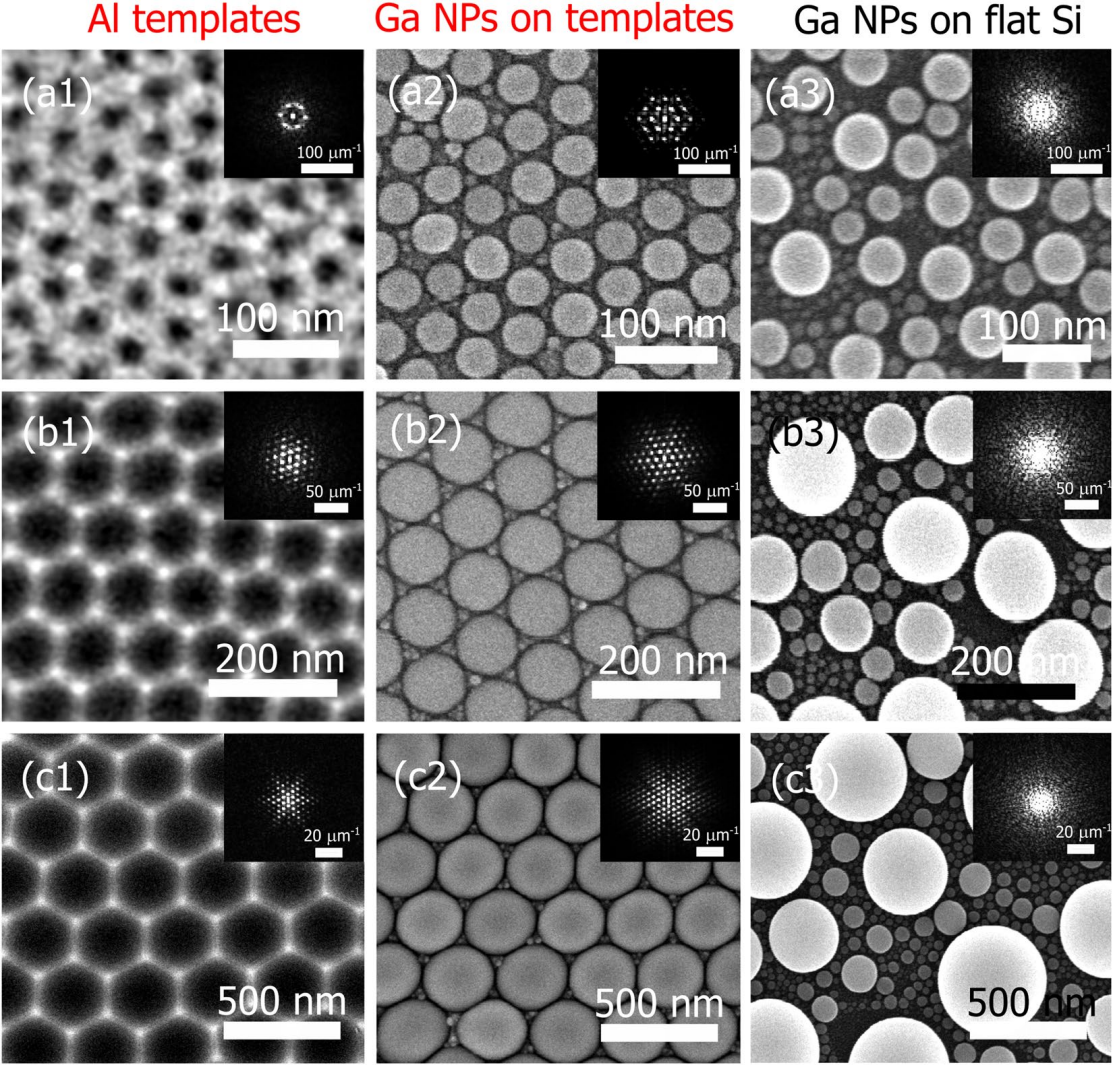
SEM images of the Al nanostructured templates of different pit diameters (first column), Ga NPs on Al templates (second column) and on flat Si (third). First row corresponds to 26 mg of Ga mass, second row to 89 mg and third row to 256 mg. The Al templates of (**a1**), (**b1**) and (**c1**) have a pit diameter of 40, 80 and 300 nm. Each SEM image has its respective FFT image in the inset.

Hexagonal ordered Al shallow pit arrays have been produced by the anodization of Al foils as described in the experimental section. Three different pit diameters have been obtained due to the different anodization conditions. Specifically, pit diameters of 40, 80 and 300 nm as shown in the scanning electron microscopy (SEM) images of Fig. 1(a1,b1,c1). A high band-pass filter was applied to the SEM images of Fig. 1(a1,b1) in order to better observe the Al pattern. The SEM images have been analysed in terms of Fourier decomposition with the Gwyddion software^54^. 2D spatial maps of Fast Fourier Transform (FFT) are represented in the insets. In all the templates (a1, b1 and c1) the dots exhibit the hexagonal pattern.

These Al nanopatterned substrates were used as templates for the ordering of the Ga NPs^53^, depositing different Ga masses in each template in order to adjust the NP size with the Al pits. The pattern of the template forces the Ga NPs to coalesce into the pits resulting in NPs with size distributions more homogeneous than those obtained on flat surfaces such as Si, sapphire or glass^18,26^.

In the second column of Fig. 1, the SEM images of the optimized cases, when NPs matches the Al pits, are shown for the three different template pit diameters (a2, b2 and c2) corresponding to Ga masses of 26, 89 and 256 mg, respectively. On the nanopatterned Al, a uniform size distribution is formed. Indeed, the FFT maps on the insets show hexagonally ordered bright dots in a long-range order, quite similar to the FFT map of the Al templates. These confirm the uniform size distributions for the Ga NPs on the three different Al templates.

In the third column of Fig. 1, the SEM images of the same Ga masses (26, 89 and 256 mg) deposited on flat Si substrates are shown for comparison. Broad size distributions are obtained in each case (Fig. 1(a3,b3,c3)) reflecting random arrangements. This is confirmed with the FFT images in the insets that do not show any remarkable feature compared to their counterparts on the Al templates.

Overall, Fig. 1 shows that a wide size range of hexagonally ordered arrays of Ga NPs can be produced by designing an appropriate nanostructured Al template^45^ and adapting the deposited Ga mass.

Once the size distribution has been improved (i.e. narrowed), we have studied the optical response of the different Ga masses evaporated by spectroscopic ellipsometry (SE). The analysis has been focused on the imaginary part of the pseudodielectric constant (<ε_2_>) since it regards the extinction response of the whole substrate-NP system, which is a good indicator of the far-field of the LSPRs^55^, as analysed later on in the simulations. The typical SE spectrum of Ga NPs on flat Si consists of two resonant bands ascribed to the two different axes in its hemispherical geometry^18,22,31,39^. The out-of-plane resonant mode due to the vertical and shortest axis of the NPs, typically observed in the UV region and the in-plane resonant mode due to the longest and horizontal axis, typically observed in the VIS-IR region.

**Figure 2:**
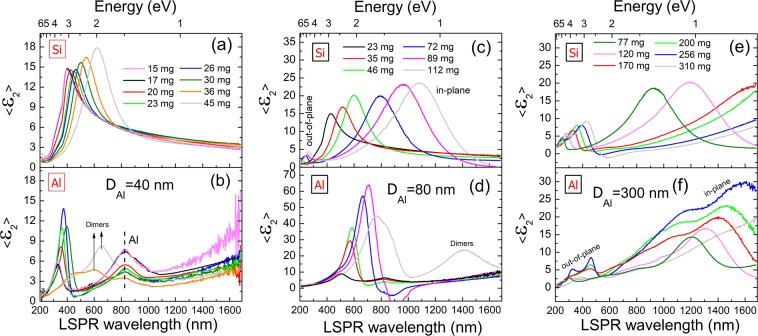
SE measurements of the set of Ga masses evaporated on the three Al templates (**b**,**d**,**f**) of 40, 80 and 300 nm of Al pit diameter (D_Al_), respectively. The counterpart Ga masses on Si are presented in (**a**,**c**,**e**).

Figure 2 shows the SE measurements of the Ga NPs for the three different templates (b, d and f) and their counterpart masses on the Si substrate (a, c and e). For the lowest pit diameter (D_Al_ = 40 nm), masses of Ga from 15 to 45 mg have been deposited. On Si (Fig. 2(a)), the LSPR wavelength is placed around 400 nm for the lowest mass. This band corresponds to the in-plane or longitudinal LSPR mode of the Ga NPs and redshifts as the Ga mass is increased. This shift is well stablished in the literature since the NP diameter is expected to be proportional to the evaporated Ga mass; whose calibration could be found elsewhere^31^. The out-of-plane mode is placed in the UV (>6 eV), out of our SE spectral range for this case. In addition to these features, the LSPR intensity increases with the Ga mass likely due to the higher scattering of bigger NPs. The SE measurements of the same Ga masses but deposited on the Al template are shown in Fig. 2(b). All of them show a band at 825 nm (1.5 eV) ascribed to the interband transition of the Al template^12^ and thus, it is not due to any plasmonic absorption. Interestingly, the LSPR intensity reaches a maximum and then decreases as the Ga mass increases. The highest intensity band (26 mg of Ga mass) matches to the most ordered Ga NPs shown previously in Fig. 1(a2). For Ga masses higher than 26 mg, the obtained NPs exceed the pit diameter and form dimers and trimers due to the coarsening of adjacent NPs^53^. As a consequence, the unimodal distribution vanishes and the LSPR intensity also falls (Fig. 2(b)). In addition, other LSPR bands appear at longer wavelengths likely due to dimers and trimers formed that are not the scope of this work.

For the intermediate pit diameter (D_Al_ = 80 nm), Ga masses from 23 to 112 mg have been deposited. The same arguments discussed before can be applied to this case. However, it is worth to note that the LSPR intensities of the Ga NPs on the Al template are much higher than on the Si substrate (the “y” axis has been set to be the double in (d) respect to (c)), suggesting a more efficient coupling. The highest LSPR intensity on the nanopatterned template is obtained for a Ga mass of 89 mg and corresponds to the SEM image of Fig. 1(b2) where Ga NPs and Al pit dimensions coincide. The most ordered cases (72 and 89 mg) shows a negative value of the <ε_2_> around 900 nm that does not have physical meaning, and is likely due to interference with the substrate as also occurred in other systems^56^ and in Ga NPs^57^. Furthermore, it is also important to mention that the out-of-plane bands appear in the UV region (200–300 nm) due to the NP size increase and redshift likewise the in-plane mode (indicated in Fig. 2(c)). This transversal mode is presented only in the SE measurements of the Ga NPs on Si and not on the Al template.

For the biggest pit diameter (D_Al_ = 300 nm), masses of Ga from 77 to 310 mg have been deposited on both substrates, Si and nanopatterned Al. On Si, the same LSPR trend is observed, redshift of both the in-plane and the out-of-plane mode (Fig. 2(e)). On the Al template the behaviour is quite similar (Fig. 2(f)). For the lowest mass (77 mg) there is a strong band at 1200 nm that redshifts and increases its intensity as the Ga mass is increased. Furthermore, the main band for each Ga mass presents at higher energies a shoulder that is likely due to higher order resonance modes such as quadrupoles based on the similarity of the NP sizes and the light wavelength in all the cases. In fact, similar Ga NP diameters have been demonstrated to support dipoles and quadrupoles modes simultaneously as evidenced by electron energy loss spectroscopy (EELS) measurements^58^. However, apart from the main band, a lower intensity bands around 200–400 nm also exist. This family of peaks belongs to the out of plane resonance mode due to different evidences: Firstly, the position of these bands agrees with the ones on Si (Fig. 2(f)). Secondly, these bands redshift as the Ga diameter increases similarly to the in-plane resonance mode^15^. Furthermore, these bands present a lower intensity than the in-plane mode and lastly, they show a shoulder at lower wavelength, which is ascribed to the quadrupole, indicating their plasmonic character. Thus, the presence of this out-of-plane resonance mode entail a certain NP eccentricity.

**Figure 3:**
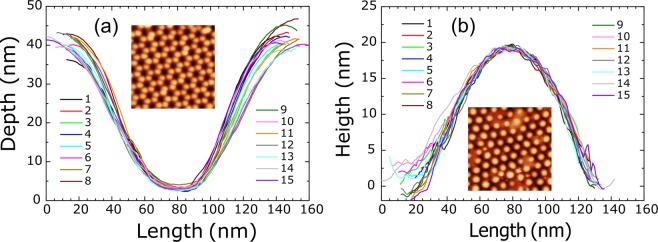
Several pit and NP profiles extracted from the AFM images, also shown as insets, of the Al template (**a**) and of the Ga NPs deposited on the template (**b**). The AFM topography image dimensions are 0.9 × 0.9 μm^2^.

Atomic Force Microscopy (AFM) has been applied to characterize both intermediate nanopatterned template and the NPs deposited on it with the purpose of reconstructing the NPs geometry. From the AFM images, profiles of the pits and the NPs have been extracted and plotted in Fig. 3(a,b), respectively, together with the topography images as insets. The pit depth and maximum NP height (apex) can be accurately interpreted since the AFM tip can access properly to those regions as it can be seen by the overlapping of the 15 profiles. However, the width of the pits and NPs is better measured by the SEM images of Fig. 1(b2) due to tip convolution effects. Thus, taking the two morphological characterization techniques, SEM and AFM, we can estimate a NP diameter of 80 nm in the horizontal axis and a NP diameter of 60 nm in the vertical axis. The eccentricity from these values correspond to ∼0.75. The same AFM procedure has been applied to the other two templates in the most ordered cases. For the lowest template we have obtained NPs of 28 nm of vertical diameter and 40 nm of horizontal diameter while for the biggest template, NPs with 213 and 300 nm of vertical and horizontal diameter, respectively. This two cases lead to aspect ratios of 0.7 and 0.71. Those values are compatible to have two different separated resonances as previously confirmed with EELS measurements^59^ and strengthen our premise that the LSPR band around 200–400 nm in Fig. 2(f) corresponds to an out-of-plane resonant mode.

**Figure 4:**
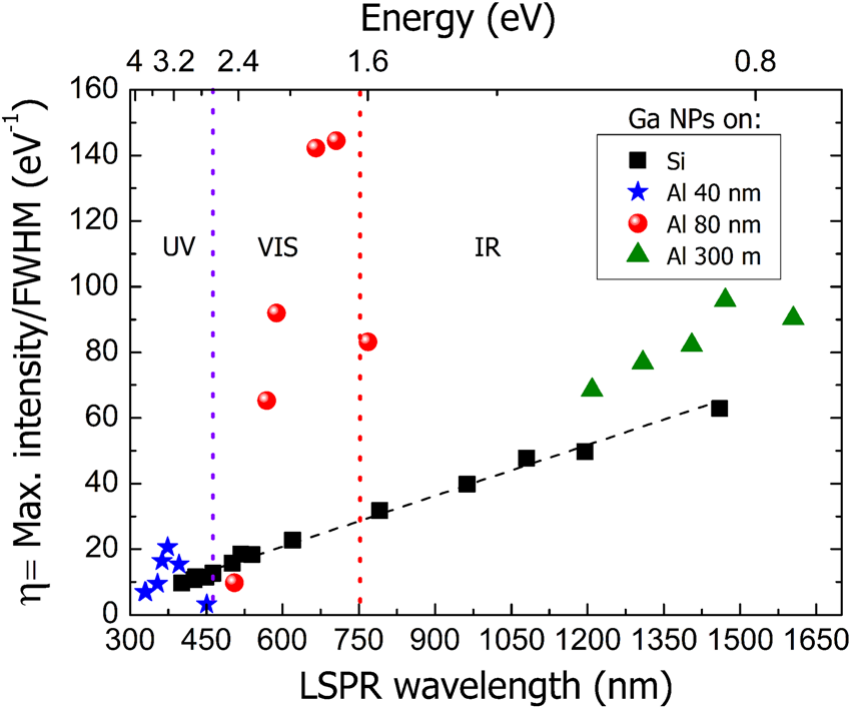
Figure of merit (*η*) as a function of the LSPR wavelength for the Ga NPs on the three different Al nanostructured templates and on Si. The figure of merit is calculated from the maximum intensity over the FWHM of the SE measurements of Fig. 2.

In order to evaluate the effect of the ordering imposed by the nanopatterning in the plasmonic properties of the Ga NPs, we have quantitatively studied the plasmonic characteristics of the SE measurements of Fig. 2. For an application point of view such as biosensing, the best scenario is to have an intense and narrow LSPR. Thus, we have calculated as a figure of merit (*η*) the ratio between the maximum intensity over the full width at half maximum (FWHM). This ratio has been represented in Fig. 4 as a function of the LSPR wavelength for the three different Al pit diameters and compared with the values obtained on Si that was the chosen substrate for our previously biosensing platforms^38,39^.

The Ga NPs on Si (black squares) show a *η* that increases with the Ga mass so as with the LSPR wavelength. This increase is likely caused by the higher scattering cross section^9^ of the bigger NPs.

The NPs on the smallest Al pit diameter (blue stars) display a *η* that reaches a maximum. The point with the highest ratio corresponds to the most ordered Ga NPs shown previously in Fig. 1(a2). The LSPR spectra of the Ga NPs on the 80 nm (red circles) and 300 nm (green triangles) Al pit diameter have also a higher *η* than the Ga NPs on Si, the optimum samples being those the SEM images of Fig. 1(b2 and, c2). Interestingly, the three optimized cases on the Al template are placed in different regions of the electromagnetic spectrum. The lowest pit diameter induces the LSPR wavelength of the Ga NPs to be in the UV, while the medium and biggest pit diameter shows their LSPRs in the VIS and IR regions, respectively, as indicated in Fig. 4. It is important to point out that the missing spectral regions in the graph between templates can be easily covered with a suitable pit diameter.

Thus, as seen in Fig. 4, it is clear that the improvement of the plasmonic features such as intensity and FWHM is directly related to the optimization of the NP size distribution. In fact, in the SE spectra of Fig. 2(b,d,f) on the Al templates the same behaviour is observed: a LSPR intensity increase and a LSPR wavelength redshift as the Ga mass increases. That mass increase means that the NPs within the pits increase in size. Thus, the more logical explanation for the peak wavelength redshift is the size increase of the NPs as it happens on Si in Fig. 2(a,c,e) and is well-known in the literature^4^. However, there is an additional factor that must be taken into account: the NP size increasing implies that NPs are closer to their neighbours what means that near-field of the NPs could interact between them causing a redshift in the far-field response. In order to numerically study this effect, we have performed DDA simulations in the far and near field of different scenarios as described in the experimental section.

**Figure 5:**
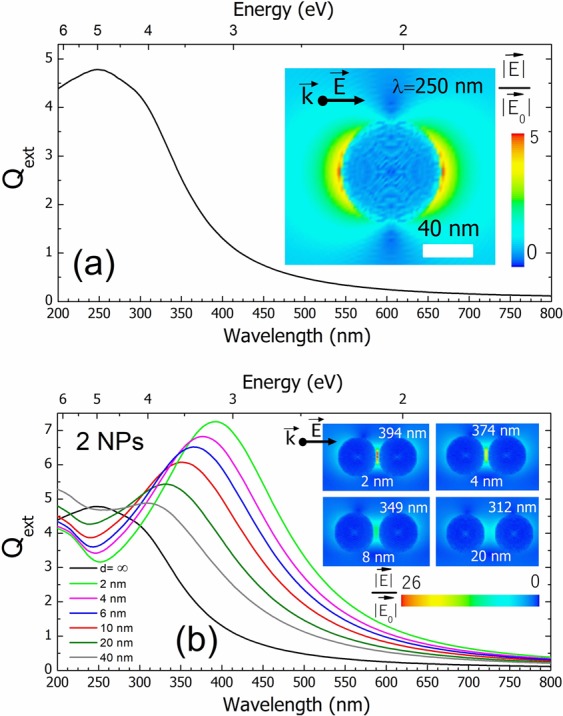
Q_ext_ analysis as a function of the wavelength and energy for two different scenarios. (**a**) A spherical single core-shell Ga NP and (**b**) a pair of Ga NPs with a variable interparticle distance. The Q_ext_ spectra of the single NP is also plotted in (**b**) for comparison. The distribution of the near electric field is illustrated in the inset of each scenario indicating the evaluated wavelength and the electric field direction. In the (**b**) inset, only the near-field for interparticle distances of 2, 4, 8 and 20 nm are shown.

The first scenario corresponds to a single spherical NP of 80 nm diameter with a native oxide shell of 2 nm similar to the ordered NP deposited on the medium template pit diameter (D_Al_ = 80 nm) of Fig. 1(b2). Figure 5(a) presents the extinction efficiency (Q_ext_) as a function of the wavelength and energy where the LSPR band is observed centred at 250 nm. The near-field has been evaluated at this wavelength and illustrated in the inset in order to observe the local electric field distribution. It shows two hot-spots in the direction of the electric field, whose intensity decays rapidly with distance in agreement with the literature^2^.

In the second scenario, we have simulated a pair of NPs of the same diameter (80 nm) with a variable interparticle distance from 2 to 80 nm. The Q_ext_ is plotted in Fig. 5(b). For an interparticle distance lower than the NP diameter, a unique band is observed in each spectrum that does not correspond to the single NP one. This new band is due to the dipolar plasmonic coupling between NPs. The phenomenon was well-described in the model by Kreibig and Vollmer^1^ and has been corroborated with experiments and simulations in several works regarding other metals^60–63^. Thus, the spectra of Fig. 5(b) reveals that in the presence of a NP close to each other, the plasmonic coupling governs the optical signal despite the single NP LSPR. Interestingly, the same behaviour for the plasmonic coupling band than in the SE spectra of Fig. 2 is observed: an increase of the intensity and a peak wavelength redshift. It means that the NP plasmonic coupling is stronger as the NPs are closer. The near-field is plotted in the inset of Fig. 5(b) for NP distances of 2, 4, 8 and 20 nm. In the case of a distance of 2 nm, the near-field image shows the most intense hot-spot in the region between NPs evidencing that the plasmonic coupling dominates, not only the far-field response, but also the near-field. In fact, a 5-fold increase of the near-field intensity is observed in the legend compared to the case of the isolated NP of the inset of Fig. 5(a). Furthermore, it can be seen in the inset images of larger interparticle distances (4, 8 and 20 nm) that the electric field intensity decreases due to the lower interaction. Indeed, the dipolar near-field of a plasmonic particle has been demonstrated to decay as the cube of the inverse distance^64^ and consequently the plasmon coupling strength becomes a function of *d*^*−*3^, being *d* the interparticle distance^65^.

**Figure 6:**
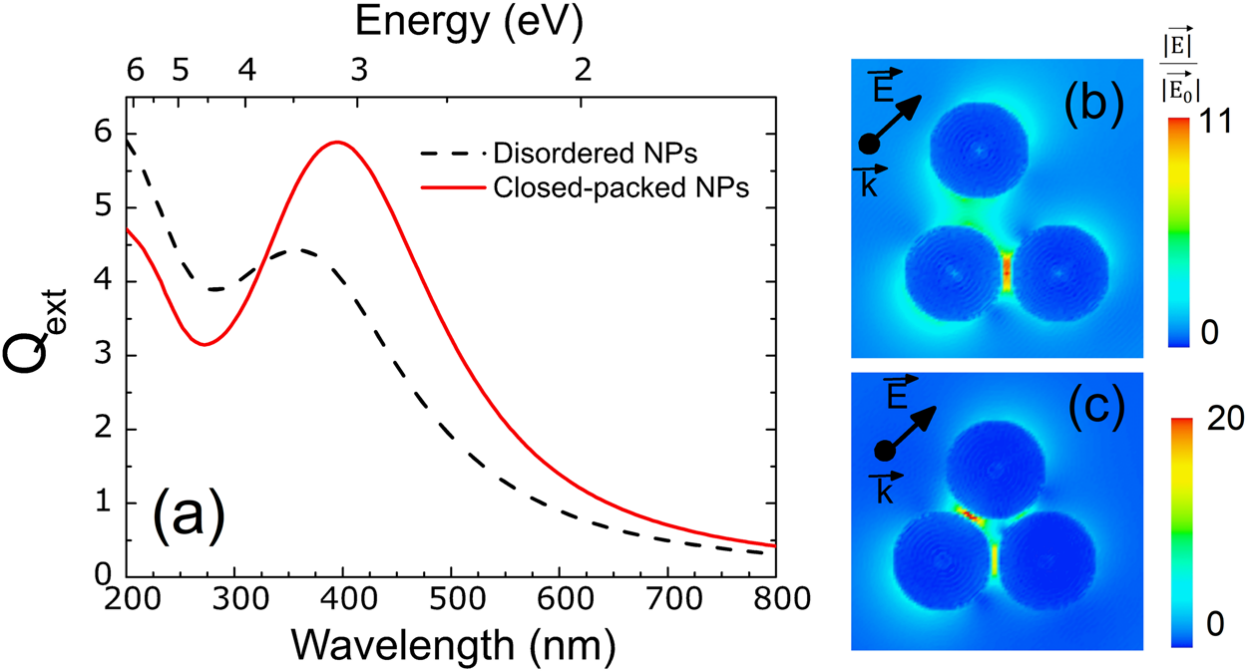
(**a**) Q_ext_ analysis as a function of the wavelength and energy for two different scenarios. A closed-packed ordered distribution of three NPs at a distance of 2 nm and a disordered distribution of three NPs. The distribution of the near electric field is also shown for the disordered (**b**) and ordered (**c**) case.

Lastly, periodicity effects have been studied with DDA simulations of three NPs that better represents the experimental size distribution obtained in Fig. 1(b2). On one hand, an ordered distribution of three NPs at a distance of 2 nm and, on the other hand, an arbitrary disordered system (Fig. 6). For the ordered case, both analysis, far and near-field, show the same results that for the two NP case simulations (Fig. 5(b)). The Q_ext_ peak wavelength is placed at the same position (394 nm); see red solid line in Fig. 6(a). Furthermore, the near-field distribution of Fig. 6(c) shows the same hot-spots between the two NPs in the direction of the electric field than the NPs pair. In the case of the disordered system, the LSPR placed at shorter wavelengths (black dashed line of Fig. 6(a)) shows lower intensity than the ordered one and its near-field distribution shows lower electric field enhancement (see the legends in (b) and (c)). In addition, the FWHM of both Q_ext_ spectra have been calculated being 240 nm for the ordered system and 259 nm for the disordered one. The LSPR of the disordered system is likely due to a convolution of LSPR bands of different intensities due to the coupling of the NPs at different distances. Note that in the experiments the scenario is much more complex with NPs surrounded by other NPs at different distances but also with different diameters as illustrated in Fig. 1(a3,b3,c3) what would lead to LSPRs with FWHM even higher than the simulated one.

Taking all the DDA simulations together, the main conclusion is that plasmonic coupling dictates the optical extinction spectra in case of two or  more NPs of the same size positioned at lower distances that their diameters. In our experimental part, the ordered NPs obtained are also spherical-like, have a similar size and their distances are lower than their diameters. Thus, it is reasonable to consider that the LSPR bands experimentally observed in the SE measurements could be due to the plasmonic coupling.

The plasmonic coupling in ordered arrays of gold and silver NPs has been deeply studied^65–68^. Indeed, the LSPR band behaviour in those works was equivalent to our experiments; an intensity increase and a wavelength peak redshift. However, these two trends can also be caused not only by the size increase but also by the decreasing interparticle distance. Thus, in order to differentiate between both factors an empirical equation was found^65^ that describes the plasmonic coupling with the wavelength shift (∆λ) as a function of the interparticle distance (*d*), but normalized with the LSPR wavelength of the isolated NP (λ_0_) and the diameter (*D*). This law named as the plasmon ruler equation has been demonstrated to be universal, since it works for several diameters, geometries, dielectric constants and materials^65^. The normalized LSPR shift (∆λ/λ_0_) decays exponentially as follows:

1 $$\frac{\Delta {\rm{\lambda }}}{{{\rm{\lambda }}}_{0}}=A\cdot exp(\frac{-d/D}{\tau })$$

being *τ* the decay rate and *A* an intrinsic factor that indicates the amplitude and is related with the analysed material.

**Figure 7:**
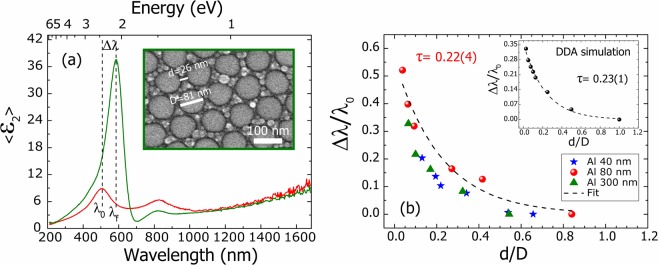
In (**a**), it is illustrated the SE measurement and SEM image in the inset were the parameters of Eq. 1 were taken for a representative case of 46 mg of Ga on the medium Al pit diameter template; ∆λ, *d* and *D* are indicated. (**b**) LSPR normalized shift (Δλ/λ_0_) as a function of the normalized interparticle distance (*d*/*D*) for the set of Ga masses evaporated on the three different Al templates. The fit of the data from the medium Al template (red circles) according to the Eq. 1 is also plotted with the obtained decay rate (τ). The inset of (**b**) shows the same plot but for the DDA calculations of Fig. 5(b) together with the fit.

In order to evaluate whether the plasmonic coupling governs our SE experimental results, we have applied this formula to the set of Ga masses evaporated on the three different Al templates. We have taken the LSPR wavelength shift (Δλ) from the SE measurements of Fig. 2. The shift is calculated from the λ_0_ that corresponds to LSPR wavelength of the isolated NP case, assumed in each template to be the SE measurement of the lowest Ga mass evaporated. The dimensions *d* and *D* have been taken from each SEM image, taking the average value from 25 different NPs. Figure 7(a) illustrates the data acquisition procedure for an arbitrary Ga mass of 46 mg on the Al template of 80 nm of pit diameter. It can be observed the Δλ obtained respect to the 23 mg Ga mass (lowest mass for this template). The inset shows the SEM image where *D* and *d* and marked. From this data, a Δλ/λ_0_ of 0.16 and a *d*/*D* of 0.27 values are obtained.

These ratios have been calculated and represented in Fig. 7(b) for each mass of the template with pit diameter of 40 nm (blue stars), 80 nm (red circles) and 300 nm (green triangles). In the three different templates, the Ga NPs LSPR normalized shift (Δλ/λ_0_) decays exponentially with the normalized interparticle distance (*d*/*D)*. We have fitted the points obtained in the template diameter of 80 nm (red circles) and obtained a decay rate (τ) value of 0.22 ± 0.04. This value matches to the reported ones by other authors in systems of nanodiscs, nanospheres and nanoparticles of Ag and Au^65,67,69^. The fit of the lowest and highest template diameter gives rise to a τ of 0.16 ± 0.02 and 0.16 ± 0.03, respectively. Despite these values fall into the data range obtained in the literature, they could be underestimated since the maximum values of the d/D are 0.55–0.65. According to the DDA simulations of the Fig. 5(b) it is necessary to have two NPs with a d/D ratio approximately around 1 to assume that there is no plasmonic coupling between them. In other words, in these cases the NPs evaporated from the lowest Ga masses are not sufficiently separated to consider them isolated in terms of plasmonic coupling and consequently the τ value obtained is likely lower than expected.

In the inset of Fig. 7(b), the LSPR normalized shift is represented as a function of the normalized interparticle distance for the DDA simulations of Fig. 5(b). The LSPR wavelength shift has been calculated from the Q_ext_ analysis as also done in the literature^65^. In this case, the points also fulfil the universal plasmon ruler equation and the τ value obtained from the fit is equal to 0.23 ± 0.01. This value coincides with the value acquired from the experimental data analysis in the same Fig. 7(b).

Thus, based on the agreement between the DDA simulations and the experimental results, it is demonstrated that the LSPRs in the ordered Ga NPs of the three different sizes are caused by the plasmonic coupling since the universal plasmon ruler equation is fulfilled. The understanding of the optical properties of these platforms will be crucial for an appropriate design of future plasmonic-based devices. Furthermore, it is worthy to note that the NP size and interparticle size are sub-wavelength in most of the cases. This fact together with the excellent arrangement and its LSPR tunability over a wide spectral range make this system a promising candidate to be a metamaterial.

## Conclusions

In summary, we have successfully produced hexagonal ordered arrays of Ga NPs of 40, 80 and 300 nm of diameter by using Al nanostructured templates. As a consequence of the ordering and the narrow size distribution, the optical response of these NPs is considerable better than the ones deposited on flat Si in terms of both, the intensity and FWHM of the LSPRs in the UV, VIS and IR regions. The origin of the LSPRs in the ordered NPs has been investigated experimentally, with the universal plasmon ruler equation, and numerically, with DDA simulations. In all the studied scenarios, the LSPR wavelength decays exponentially with the interparticle distance, being the decay rate around 0.2 in agreement with the literature. These results confirm the plasmonic coupling as the cause of the LSPRs in the ordered NPs. The enhanced optical performance due to the improvement of the NP size distributions and its comprehension will contribute to the development of applications based on these ordered NPs such as biosensors.

## Experimental and Simulation Methods

### Growth of the nanopatterned template

Al nanopatterned substrates of different pit diameters were used as templates for the ordering of the Ga NPs. The patterned templates were prepared on high-purity Al foils (99.999%) of 0.5 mm of thickness and 25 mm of diameter by anodization process. The experimental conditions of the anodization depend on the desirable pit diameter^52^. In this work, three different pit diameters have been used whose growth procedure is described as follows.

For a 40 nm pit diameter the anodization was done for 16 h in sulphuric acid electrolyte (2.15 M) under 20 V constant voltage and constant temperature between 0 to 1 °C^70^. Due to this process, AAO pores are formed also known as alumina membrane. Since our aim is the Al nanostructure below the alumina, the growth pores are chemical etched by immersion in a mixture of 0.18 M chromic oxide (H_2_CrO_4_) and 0.72 M phosphoric acid (H_3_PO_4_) for 24 h. The result is a hexagonal Al array of ordered shallow pits as shown elsewhere^53^.

For 80 nm pit diameter: The Al is anodized for 24 h in oxalic acid electrolyte (0.3 M) under 40 V constant voltage and constant temperature of 3 °C^71^. Then the AAO is removed with the same selective chemical etching described before.

For a 300 nm pit diameter: The hard anodization^72,73^ is done in oxalic acid electrolyte (0.3 M) with a 5% of ethanol at a constant temperature of 0 °C. Firstly, at a voltage of 80 V during 900 s in order to create a protective Al oxide layer that prevents the subsequent rupture. Then, the voltage is increase up to 130 V at a rate of 0.08 V/s and keep there for 3600 s. After the anodization, the AAO is removed.

### Ga deposition

The Ga NPs were grown by Joule-effect thermal evaporation. The process takes place at a base pressure of 2 × 10^−7^ mbar in a vertical Edwards E306 vacuum chamber. Ga (purity of 99.9999%) is evaporated in a tungsten filament (99.90% purity) at a power of 50 W. The Al template samples as well as Si (100) reference samples were placed 200 mm away from the Ga source. The size of the Ga NPs is controlled by the Ga mass added to the crucible as shown elsewhere^31^. In this work, the Ga mass has been varied from 15 mg to 310 mg that produces NPs with diameter from 20 to 400 nm.

### Optical and morphological characterization

After the Ga NPs growth, the optical properties of the samples on the Al template and on the reference Si were measured by SE using a Woollam M-2000 ellipsometer (J.A. Woollam Inc). The measurements were taken at an incident angle of 75° within the spectral range from 200 to 1700 nm. The analysis was focused in the imaginary part of the pseudodielectric constant (<ε_2_>) with the aim to compare with the extinction efficiency of the latter simulations. <ε_2_> was obtained from the ellipsometric parameters psi (ψ) and delta (Δ)^74^.

The morphology of the Ga NPs and the Al templates was analysed by SEM and AFM. The SEM is a FEI XL30-SFEG system, operating with 10 keV electron beam and nominal lateral resolution of 4 nm, being the secondary electrons collected and analysed with an Everhart-Thornley detector. The AFM is an Agilent PicoPlus 5500 system. The topographical images were obtained with Si cantilevers whose tips have a nominal radius of 8 nm and force constant of 40 N/m (Brucker) working in dynamic mode. Images were analysed and post-processed with the Gwyddion software^54^.

### Discrete dipole approximation simulations

In order to study the origin of the LSPR band observed by SE with numerical calculations, we have carried out simulations with the DDA method with the DDSCAT 7.2. code^75^. The three scenarios used in these simulations were created by a target generation tool program executed in Matlab^76^: a single core-shell spherical Ga NP, a pair of NPs with a variable interparticle distance and an array of three NPs at a distance of 2 nm. Liquid Ga surrounded by its protective oxide shell (Ga_2_O_3_) constituted the core-shell structure. The diameter of the NPs was chosen to be 80 nm being the oxide thickness equal to 2 nm in order to represent the NPs with the best experimental results. A dipole lattice spacing of 2 nm was used in all cases, giving 33401 dipoles for the isolated NP. The error tolerance for convergence of the calculations was set to 10^−5^ at each wavelength^77^. The dielectric constants of the Ga and Ga_2_O_3_ materials were obtained from the literature^78,79^. The analysis was focused in the Q_ext_ calculated from the ratio of the extinction cross-section and the geometrical cross section.
